# How do it: tips and tricks during full-robotic pancreaticoduodenectomy

**DOI:** 10.1007/s13304-025-02264-4

**Published:** 2025-05-25

**Authors:** T. Piardi, G. Badessi, S. A. Biondo, C. Del Basso

**Affiliations:** 1 Simone Veil Hospital, UniversityofReimsChampagne-Ardenne, Troyes, France; 2 CHU Reims, UniversityofReimsChampagne-Ardenne, Reims, France; 3 Policlinico G. Martino, University of Messina, Messina, Italy

**Keywords:** RPD, Pancreaticoduodenectomy, Robotic pancreaticoduodenectomy

## Abstract

Robotic pancreaticoduodenectomy (RPD) is increasingly recognized as a standard procedure due to its minimally invasive nature and associated benefits such as reduced blood loss and faster recovery. This paper presents a set of tips and tricks, focusing on the management of pancreatic and biliary sections, the application of the falciform ligament for vascular protection, and the precise positioning of drainage systems. Key strategies include the use of 4/0 PDS stitches to control pancreatic vessel bleeding, preventing ischemia by refraining from the use of energy during pancreas dissection, and ensuring optimal drainage placement. The incorporation of a modified Blumgart anastomosis with a single-thread technique and the use of hydrogel sheets are also highlighted as crucial in reducing fistula formation. We think that these measures can significantly lower the incidence and severity of POPF and postoperative bleeding, thereby enhancing surgical outcomes. This paper aims to contribute to the growing body of knowledge on RPD, offering practical tips that can be safely and effectively integrated into routine clinical practice.

**Supplementary Information:**

The online version contains supplementary material available at 10.1007/s13304-025-02264-4.

## Introduction

Since the advent of robotic platforms in pancreatic surgery, the use of robots for performing pancreatico-duodenectomy (PD) has increased significantly. Compared to laparoscopy, robotic pancreatico-duodenectomy (RPD) can beneficiate of the improved 3D vision, the enhanced dexterity of robotic instruments in a stable platform that can overcome limits of laparoscopic technique when fine dissection is needed and during the reconstruction phase.

However, pancreatic surgery continues to be associated with high morbidity and mortality rates, primarily due to its surgical complexity and the elevated risk of postoperative pancreatic fistula (POPF). By now, several major reports failed to show the superiority of robotic PD on open technique with the incidence of severe complications similar between the two groups [1, 2].

Various technical strategies have been proposed to mitigate potential surgical complications (SC), such as post-operative pancreatic fistula (POPF) or hemorrhage, which are associated with morbidity, prolonged hospital stays, and mortality—even in high-volume centers.

In this technical note, we present key tips and tricks derived from our experience in pancreatic surgery, which we have applied in our most recent cases of fully robotic distal pancreatectomy (DP).

### Cases series

The patient is positioned in the standard setup for robotic pancreaticoduodenectomy (RPD), as shown in the video, with the optical trocar placed two fingerbreadths left lateral to the umbilicus. The placement of the robotic trocars is determined by the patient’s abdominal size, while maintaining the umbilicus as a reference point.

### Stiches to prevent bleeding before pancreatic section

To reduce anticipated bleeding from the superior and inferior pancreatic vessels, we propose placing four PDS 4/0 stitches—two on the superior and two on the inferior borders of the pancreas. The stitches on the superior margin should be full-thickness, securing at least 1 cm of pancreatic parenchyma. This technique, particularly beneficial in cases of a soft pancreas, may help to reduce hemorrhage (as showed in the video) from pancreatic subcapsular vessels during transection. Moreover, uncontrolled bleeding may necessitate the use of thermal energy on fragile tissue, potentially leading to suboptimal coagulation and delayed hemorrhage.

### Pancreatic section

We transect the pancreatic duct using “cold” scissors, without systematically applying any form of energy to the instrument. Coagulation is used only when cutting the pancreatic parenchyma, ensuring it is applied at a safe distance from the pancreatic duct. This approach is recommended to minimize the risk of ischemia to the duct. This technique has been described by our surgical team [3].

### Biliary section

Whenever possible, the bile duct should be transected as late as possible during the resection phase of the procedure. To minimize capillary bile leakage, particularly in cases with an intra-biliary stent, the bile duct is typically clamped using a bulldog forceps. However, in cases where the bile duct has thin walls, we place a resorbable hemostatic patch inside the biliary lumen to prevent ischemic injury to the wall, which could lead to a post-operative biliary fistula.

### Falciform ligament around the vessels

One crucial step before reconstruction, following lymphadenectomy, is protecting the arterial vessels from direct exposure to active pancreatic juice, which is a common cause of visceral pseudoaneurysms. Various techniques have been described in the literature, though many are challenging to implement in robotic distal pancreatectomy (DP). Based on our experience, we utilize the falciform ligament to wrap around the hepatic artery and gastroduodenal stump, as described in the PANDA trial [4].

This technique not only provides complete arterial coverage but also helps"fill"the space between the celiac trunk origin, the anterior wall of the vena cava, and the left hepatic margin—an area frequently prone to fluid collection. The falciform ligament wrap is secured using two to three interrupted Prolene 5/0 stitches or a continuous V-Lok 5/0 suture.

### Position of the drains

A well-functioning drainage system is essential for pancreatic anastomosis. Our team utilizes a non-aspirative drainage approach, with drains positioned before completing the modified Blumgart anastomosis and choledochojejunostomy. Placing the drains at this stage ensures optimal positioning to effectively manage potential leaks from the anastomoses.

We recommend placing the drains before finalizing all anastomoses, as correct placement afterward may be challenging and could create unintended traction on the anastomoses. We use a multi-tubular drain with three distal branches:

- The upper branch is positioned just beneath the future choledochojejunostomy and anterior aspect of the pancreatic anastomosis.
- The middle branch is placed under the future pancreaticojejunostomy.
- The lower branch is positioned laterally to the pancreas.

This strategic placement helps optimize drainage while minimizing the risk of anastomotic tension or interference.

### Pancreatic anastomosis

**Fig 1:**
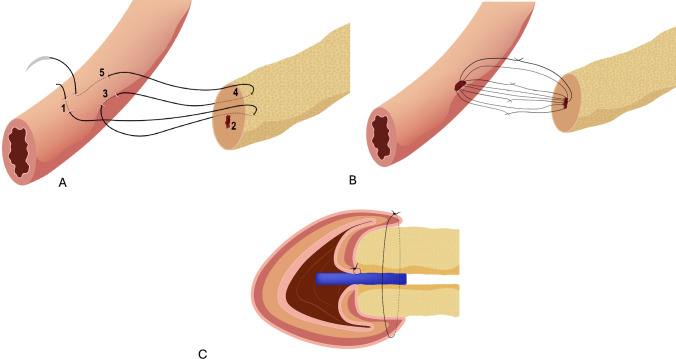
full robotic Blumgart pacreatico-jejuno anastomosis modified according to Reims. **a** 4/0 V-Lok (single needle) anastomosis with thread tied on the jejunal side **b** ducto-mucosa anastomosis 5/0 pds 2 or 4 stitches at the edges of the wirsung according to the diameter **c** lateral view of the anastomosis with intra wirsung prosthesis in place

The technique we use, previously presented by our team, is similar to a modified Blumgart approach, it basically involves a duct-to-mucosa anastomosis combined with transpancreatic U-sutures to invaginate the pancreatic parenchyma. The key steps of this anastomosis, as demonstrated in the video, include: placement of transpancreatic U-sutures—using a single-needle, non-absorbable or long-lasting absorbable suture (V-Lok 4/0 or Prolene 4/0 with a 24 G needle), the first suture is placed through the anterior seromuscular layer of the jejunum. The needle then passes through the full thickness of the pancreatic parenchyma (anterior-posterior), avoiding injury to the main pancreatic duct. It is then passed unto the posterior wall of the jejunum (Fig. 1a). It is then brought back through the anterior pancreatic capsule and the anterior seromuscular layer of the jejunum, creating a U-shaped transpancreatic mattress suture. Knot placement: the knot is tied at the edge of the intestine rather than on the pancreatic wall. Depending on the length of the pancreatic stump, we place a total of 2 or 3 sutures. Hydrogel patch incorporation: in case of soft pancreas, during the passage of the suture through the pancreatic stump, a previously positioned hydrogel sheet patch (Neovil^®^) is integrated into the anastomosis, wrapping around the stump like a scarf. Duct-to-mucosa anastomosis (inner layer): a small enterotomy is made in the jejunum at the site of the anastomosis. The enterotomy is tailored to match the size of the pancreatic duct. Using fine monofilament sutures (5/0 PDS or Prolene), a precise duct-to-mucosa anastomosis is performed with two to four interrupted sutures. The stitches are placed between Wirsung duct and tailored jejuno-enterotomy. We use to put a 6-cm endoscopic stent with a leading barb, inserted before securing the surgical knots. If the Wirsung duct diameter is ≤2 mm, the stitches are unnecessary, as the stent alone is positioned and held in place by the leading barb.” Technical steps are showed in Fig. 1. Completion of the transpancreatic sutures: the previously placed U-sutures are tied gently to secure the pancreas to the jejunum. These transpancreatic sutures reinforce the anastomosis and reduce tension on the duct-to-mucosa sutures. Final hemostasis and vascular assessment: we assess pancreatic stump vascularization using indocyanine green (ICG). ICG is administered as a bolus at 0.1 mg/kg according to institutional protocol. The fluorescence pattern of the pancreatic stump is evaluated on the free side of the anastomosis, as shown in the video. Homogeneous fluorescence indicates good vascularization of the anastomosis [5].

## Results

In 2023, we began implementing all these recommendations collectively during our robotic pancreaticoduodenectomy (RPD) procedures, applying them to a total of 13 patients. The surgical indications included: 8 cases of pancreatic ductal adenocarcinoma (PADC), 1 case of duodenal cancer, 2 cases of suspected intraductal papillary mucinous tumor (IPMT) degeneration, 2 cases of distal cholangiocarcinoma. Postoperatively, we observed: 1 case of grade B post-operative pancreatic fistula (POPF) (7.6%), 1 case of post-operative hemorrhage (7.6%), both successfully managed with radiological intervention. There was no post-operative mortality.

Additionally, in 20% of cases, we noted a transient increase in serum amylase and lipase levels, defining a PPAP—post pancreatectomy acute pancreatitis grade 1 [6]—likely due to pancreatic"irritation"caused by the endoscopic stent in the pancreatic duct. Routine CT scans performed on postoperative day 7 (J7) in these patients confirmed pancreatitis signs [6].

## Conclusion

This technical note provides a set of tips and strategies for trying to minimize surgical complications during full robotic pancreaticoduodenectomy (PD), with a focus on managing the pancreatic and biliary sections. It also emphasizes the use of the falciform ligament for vascular protection and the careful positioning of drainage systems. Key techniques include the application of PDS 4/0 sutures to control bleeding from pancreatic vessels and a modified Blumgart anastomosis.

## Supplementary Information

Below is the link to the electronic supplementary material.

Supplementary file1 (MP4 409509 kb)

## Data Availability

Not applicable.
